# A Conservative Approach to Surgical Management of Root Canal Perforation

**DOI:** 10.1155/2021/6633617

**Published:** 2021-01-20

**Authors:** Régis Augusto Aleixo Alves, André Luiz Gomide Morais, Thábata Frederico Izelli, Cyntia R. A. Estrela, Carlos Estrela

**Affiliations:** Department of Stomatology Sciences, Federal University of Goiás, Goiânia, Brazil

## Abstract

This study describes a conservative approach to surgical management of root canal perforation in maxillary lateral incisors. A patient was referred for retreatment of a maxillary lateral incisor. Her chief complaint was discomfort in the buccal mucosa. Periapical radiography showed radiopaque material consistent with sealing material inside the root canal. A CBCT scan was acquired and revealed a gutta-percha cone outside the root canal, from the middle third to beyond the root apex. The imaging examination showed that the pulp cavity had not been affected. Thus, we took the clinical alternative of surgically managing the perforation by sealing with MTA, thereby avoiding endodontic treatment, and followed up with only clinical and radiographic control. At the two-year follow-up, after the surgical procedure to remove the extruded filling material, we observed bone tissue formation and positive response to pulp tests, without any clinical signs or symptoms. Root perforation is considered an unpleasant error in an operative procedure. Once a perforation is properly diagnosed, located, and sealed with biomaterial, a favorable prognosis is often achieved. MTA offered good sealing of the perforation, with promising results. Decision-making using the CBCT scan enabled us to adopt a conservative approach and favored more reliable treatment predictability.

## 1. Introduction

Root perforation results in communication between the root canal system and the external tooth surface [1]. When completing the last steps of the endodontic therapeutic protocol, all care must be taken to avoid accidents that may risk losing teeth [2].

In clinical practice, pathological perforations are frequent. Iatrogenic root perforations may occur at any time in root canal treatment, during access cavity opening, root canal preparation, or post preparation. All these procedural operative errors may lead to treatment failure [3–6].

In this respect, previous planning for root canal treatment becomes essential, particularly clinical and radiographic examination. Operative procedures prior to access cavity preparation involve removal of all carious tissue, restoration of defects, and weakening of the dentin structure, actions which could change the coronal references. Careful analysis of the coronary chamber based on 3-dimensional imaging exams, well-planned selection of a drill compatible with the coronary volume, and good lighting and magnification are essential procedures, because they favor visualization of the cavity during coronary opening, and prevent unpleasant accidents [2, 6].

The advent of new technologies incorporated into imaging exams in endodontics, such as cone beam computed tomography (CBCT) [7–9], have impacted the outcome of root canal treatment. Better diagnostic accuracy across several clinical conditions [8–12] and better predictability in the decision-making process in clinical practice have allowed establishing more conservative therapeutic protocols. In this respect, the present study describes a conservative approach to surgical management of root canal perforation in a maxillary lateral incisor.

## 2. Case Presentation

An 11-year-old female patient in good general health sought treatment at the Public Dental Specialty Center, Brazil, with the chief complaint of mild discomfort in the buccal mucosa of the maxillary lateral incisor. The patient and the caregiver reported that endodontic treatment was carried out on this tooth 1 year prior, owing to a history of dental traumatism with coronary fracture.

**Figure 1:**
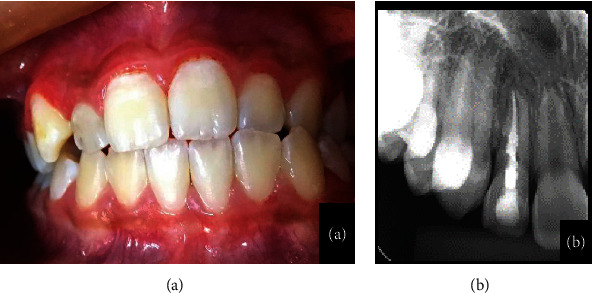
(a, b) Buccal gingival tissue of the maxillary lateral incisor reveals normal clinical features. Initial periapical radiography shows sealing material in the pulp cavity.

**Figure 2:**
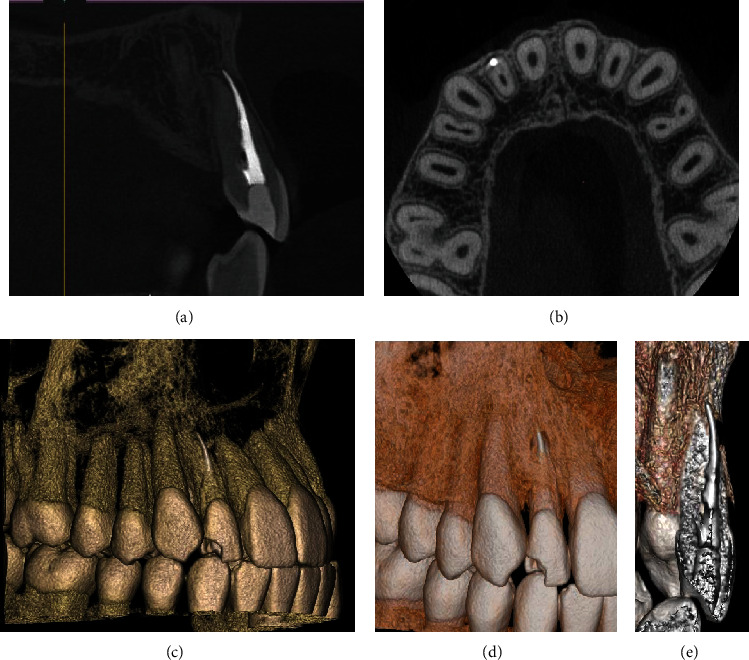
CBCT scans (a–e) and 3D reconstructions show sealing material out of the channel, extruded by root perforation in the middle third of the root canal.

**Figure 3:**
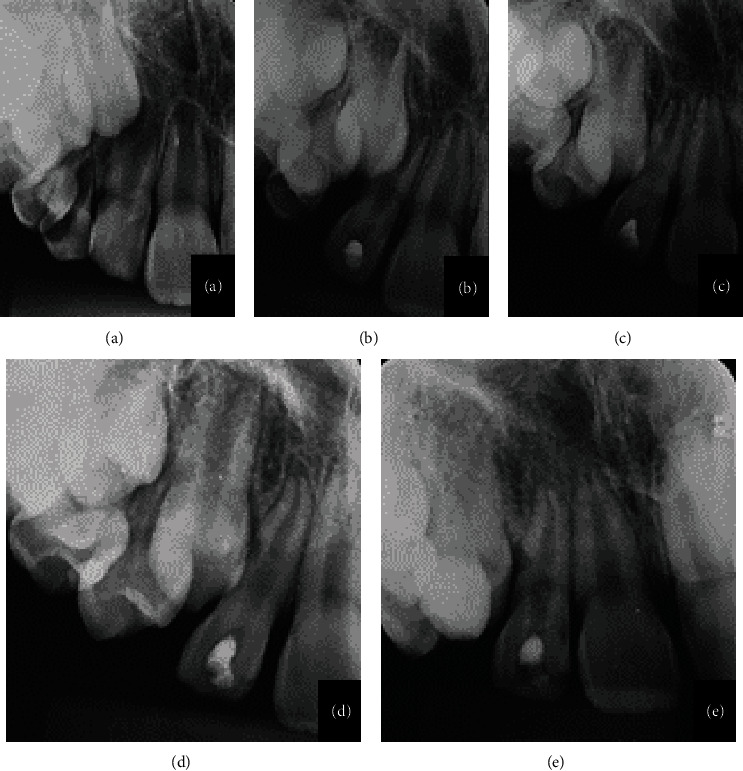
(a–e) 26-month radiographic follow-up: periapical radiography before endodontic access and root perforation (a); maxillary lateral incisor with evidence of coronal access (b); follow-up at 14 (c) and 20 (d) months; evidence of complete rhizogenesis (e).

Clinical examination revealed no signs of swelling, fistula, or change in the tooth color (Figure 1(a)). No pain was manifested in the percussion tests, but moderate pain was reported during palpation of the buccal mucosa. Pulp tests were performed in all maxillary anterior teeth using Endo-Frost (Roeko-Wilcos, Rio de Janeiro, RJ, Brazil), which suggested the presence of vital pulp tissue, including tooth #7. The periapical radiography revealed no periapical radiolucency and showed filling material apparently inside the root canal, a situation incompatible with the positive response of the pulp vitality test (Figure 1(b)). Thus, a cone beam computed tomography scan (PreXion, San Mateo, CA, USA) was obtained to improve image interpretation and achieve a more reliable diagnosis. Both sagittal and axial planes revealed a root perforation in the middle third of the buccal surface, with no evidence of invasion of the root canal space. In addition, the tooth showed signs of normality, with complete closure of the root apex and without apical periodontitis, characterizing the physiological process of rhizogenesis (Figures 2(a)–2(e)). Seeking to ensure that the most appropriate decision would be made, the colleague responsible for the case was asked to provide us with all the radiographs taken between the start of the treatment until the supposed root canal filling. Our request was promptly met and allowed us to perform the chosen surgical technique more confidently. Previous radiographs requested of the patient revealed that the rhizogenesis process was evolving naturally without any complications, despite the root perforation. One hypothesis is that the first professional who started treating the incomplete rhizogenesis saw that the root apex was completely closed and concluded the endodontic treatment without realizing that the sealing he had performed was actually outside the root canal. Radiographic follow-up over time revealed that the root had developed naturally, given that the pulp tissue was healthy (Figures 3(a)–3(e)).

Based on the radiographic aspect of the patient's history, and the favorable condition of pulp vitality, a conservative surgical approach was considered the best choice. Surgery was limited to removal of the extruded gutta-percha and subsequent sealing of the root perforation without any endodontic intervention.

**Figure 4:**
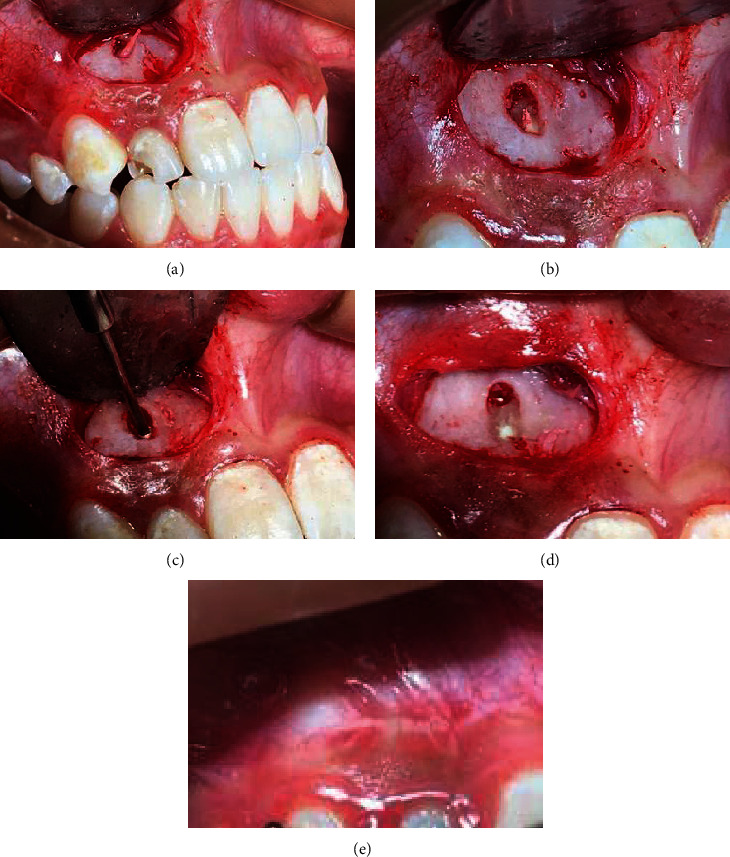
(a–e) Surgical procedure for removal of extruded gutta-percha and sealing with MTA; one year after the surgery, showing the gingival tissue in the buccal area, with normal characteristics.

**Figure 5:**
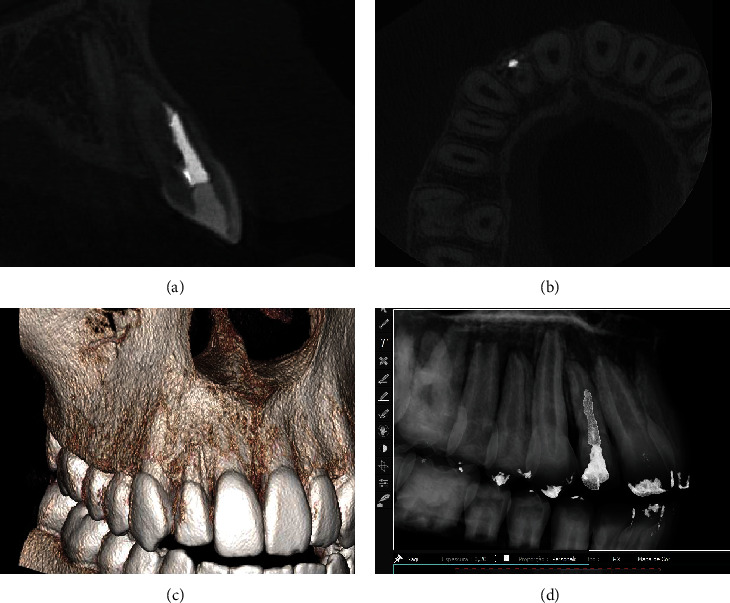
(a–d) CBCT scan and 3D reconstructions two years after the surgery show healing process of the buccal bone and absence of signs of inflammation in the root apex.

Once the treatment plan was established, the patient signed an informed consent form agreeing to undergo the study procedures. A 2% lidocaine solution containing 1 : 100000 adrenaline (DFL, Rio de Janeiro, RJ, Brazil) was administered as local anesthesia for the right infraorbital nerve, followed by the surgical phase, initiated by making a semilunar incision. After the surgical flap was made, the root perforation was located, and the extruded gutta-percha was exposed (Figures 3(a) and 3(b)). Removal of the extruded gutta-percha was performed with heat condensers, and the cavity was sealed with white MTA (Angelus Ltda, Londrina, Brazil) (Figures 4(a)–4(e)). The suturing was done with nonabsorbable monofilament nylon. The final aspect of the radiographic exam conducted immediately after surgery may cause confusion, because it suggests that the canal was filled below the working limit, owing to the limitations of two-dimensional exams. After seven days, the patient returned to remove the suture, and 10 days following the surgery, the mucosal wound healed completely. In a subsequent follow-up two years after the periapical surgery (Figures 5(a)–5(d)), a new CBCT scan was obtained, and bone repair was observed in the root perforation area, as well as aspects of normality at the periapical level (Figure 3(e)). Clinically, the patient remains asymptomatic with no signs of inflammation in the gingival mucosa and is responding positively to the pulp vitality tests. The patient was informed of the required continuation of follow-up and control appointments.

## 3. Discussion

Root perforations may occur at any time during root canal treatment and require preventive measures, so that unpleasant accidents of this nature can cease to become a risk factor for tooth loss. The consequences of root perforation may result in an inflammatory response associated with periodontal tissue and alveolar bone destruction [6].

Diagnosis of the perforation is usually made by clinical investigation and imaging exams. After the advent of cone beam computed tomography (CBCT), anatomical alterations and pathological conditions not identified by way of conventional radiographic examinations became clearly evidenced and could be measured by volumetric study, a feature offered by CBCT. The accuracy in detecting apical periodontitis using CBCT is significantly greater than that of panoramic and periapical radiographs [9–12].

Periapical radiography offers information on two planes and cannot perform three-dimensional reconstruction. This represents an important limitation that can compromise decision-making and prognosis, mainly in cases of buccal and lingual root perforation, in which overlapping structures can hide the pathological area.

Root perforation caused by an operative procedure error represents the vast majority of cases and may be associated with lack of professional ability and factors related to anatomy and dental position, the presence of pulp calcifications, extensive caries, and the presence of intracanal posts [6, 13, 14]. The prognosis is related to treatment time and the size and location of the perforation. The success rates are higher in situations of immediate repair or cases of small perforations located in the coronal or apical thirds [3, 4, 6].

Given these characteristics, the prognosis for this clinical case would have been unfavorable, since the iatrogenic procedure was caused by a drill driven in high rotation in the middle third of the root, and the perforation was sealed only years later. However, after a two-year follow-up, the prognosis was very favorable, especially considering the positive response of pulp vitality. The good response seen in this case may have been related to the patient's age and the great pulp regeneration capability of immature teeth [15], associated with conservative management.

In the present clinical case, the conservative management of the root perforation, by avoiding the conventional treatment of the root canal, allowed the root to develop without any complications. Furthermore, image monitoring showed that the root was developing according to the classic pattern of physiological apical closure (continuity of longitudinal and transversal growth of the root without decreasing the size of the tooth) (Figures 2(a)–2(e)). This is unlike cases treated with apexification (resulting in less thick dentin walls and apical closure induced by intracanal medication and ultimately producing reduced root dimensions) [13–16].

In regard to the imaging tests, the use of CBCT scans has brought numerous benefits to endodontics. Because CBCT is a dynamic examination, and images can be viewed in axial, sagittal, and coronal sections, the possibility of navigation represents an excellent resource that significantly improves diagnosis and planning [7–12]. This type of technology has become quite common among endodontists, especially in cases of nonsurgical retreatment and presurgical planning, since it saves time and effort during canal treatment procedures, by searching for canals or discovering why a previous treatment has failed [2, 17].

However, the production of beam hardening artifacts can hinder the interpretation of the images and consequently compromise the diagnosis and decision-making in complex cases. More recently, software packages have been developed [8] to aid in resolving or at least in reducing these limitations observed in viewing the exams. The e-Vol DX (CDT, São José dos Campos, Brazil) is a new CBCT software package capable of changing volume parameters, such as thickness and slice intervals, correcting data, applying image filters, and manipulating brightness and contrast. Compared to other software packages, its main advantages include compatibility with all current CBCT scanners, ability to export DICOM data, access to a more comprehensive brightness and contrast library, customized slice thickness adjustment, adjustable custom sharpness, advanced noise reduction algorithm that improves image quality, predefined image filters, dedicated endodontic volume rendering filters able to enlarge the image by 1000x (3D reconstructions) without loss of resolution, and automatic customization of image parameters for better standardization and improved research procedures [8].

In this clinical case, the CBCT images were crucial in determining the suitable diagnosis and establishing a favorable interventive surgical plan. The sealer material appeared inside the root canal in the periapical radiography but was found to have been extruded by root perforation on the buccal surface of the tooth in the CBCT images, with destruction of the bone cortex. Measurements of the exact location of the root perforation could be made, thus enabling a conservative surgical approach to be performed with minimal damage to the gingival and bone tissues, and ensuring a better postoperative outcome and favorable prognosis for the patient. In the two-year follow-up, e-Vol DX software tools enabled viewing the repair in great detail at the surgical intervention site.

Root perforation represents a significant iatrogenic error, committed especially by professionals less experienced in endodontics, but is a procedural error that may also be made by professionals with greater expertise. Anomalies in dental development such as C-shaped canals, *dens invaginatus*, taurodontism, root fusion, root dilaceration, giro version, microdontia, and palatogingival grooves, as well as physiological endodontic conditions, such as calcified/curved canals and pulp stones, are factors that may jeopardize the endodontic treatment and make it susceptible to iatrogenic maneuvers [2, 17].

## 4. Conclusions

The use of modern technological tools and professional updating are attitudes that contribute to improving diagnosis and decision-making and that characterize the recommended conduct for both prevention and management of procedural errors, to ultimately offer better predictability of root canal treatment.

## Data Availability

The data supporting this study can be accessed by readers freely through the availability of the same by the authors, as long as the patient's personal data is preserved. Images can be requested and notes from the medical record can also be viewed at any time, as long as there is no identification of the patient, as previously stated.
